# US state-level containment policies not associated with food insecurity changes during the early COVID-19 pandemic: a multilevel analysis

**DOI:** 10.1017/S1368980024002696

**Published:** 2025-01-23

**Authors:** Samantha M Sundermeir, Erin Tigue, Francesco Acciai, Emma Moynihan, Meredith T Niles, Roni Neff

**Affiliations:** 1 Department of International Health, Johns Hopkins University Bloomberg School of Public Health, Baltimore MD, USA; 2 Johns Hopkins Center for a Livable Future, Johns Hopkins University, Baltimore, MD, USA; 3 College of Health Solutions, Arizona State University, Phoenix, AZ, USA; 4 Department of Environmental Health & Engineering, Johns Hopkins University Bloomberg School of Public Health, Baltimore, MD, USA; 5 Department of Nutrition and Food Sciences and Food Systems Program, University of Vermont, Burlington, VT, USA; 6 Gund Institute for Environment, University of Vermont, Burlington, VT, USA

**Keywords:** Food security, Containment policies, Stringency Index, COVID-19

## Abstract

**Objective:**

To investigate the relationship between US containment measures during the COVID-19 pandemic and household food insecurity.

**Design:**

To investigate these relationships, we developed a framework linking COVID-19-related containment policies with different domains of food security and then used multilevel random effects models to examine associations between state-level containment policies and household food security. Our framework depicts theorised linkages between stringency policies and five domains of food security (availability, physical access, economic access, acceptability in meeting preferences and agency, which includes both self-efficacy and infrastructure). We used US national data from a representative survey data from the National Food Access and COVID research Team that was fielded in July–August 2020 and April 2021. Containment policy measures came from the Oxford Stringency Index and included policies such as stay-at-home orders, closing of public transit and workplace closures.

**Setting:**

The USA.

**Participants:**

3071 adult individuals from the National Food Access and COVID research Team survey.

**Results:**

We found no significant associations between state-level containment policies and overall food insecurity at the state level or any of the individual domains of food insecurity.

**Conclusions:**

This research suggests that while food insecurity across all domains was a significant problem during the studied phases of the pandemic, it was not associated with these containment measures. Therefore, impacts may have been successfully mitigated, likely through a suite of policies aimed at maintaining food security, including the declaration of food workers as essential and the expansion of federal nutrition programmes.

Following the onset of the COVID-19 pandemic in early 2020, governments across the globe adopted various levels and types of containment policies to control infection. The most stringent restrictions in the USA were observed in early April 2020^(1)^. These regulations simultaneously impacted both the supply and demand sides of the food system and led to individual job and financial losses and mobility restrictions, as well as disruptions in food supply chains resulting from indirect impacts from restrictions on workers and food trade^(2–4)^. During the early months of the pandemic, a marked spike in food insecurity rates was observed across the USA^(2,3)^. The nature of the relationship between containment policies and food security has not been explored. This article presents a conceptual framework describing how pandemic containment policies might affect diverse domains of household food security and uses multilevel analysis to assess the association between state-level containment policies and domains of household food security in the USA.

## Dimensions of food security during the pandemic

Food insecurity is defined as a lack of consistent access to enough food for an active, healthy life^(5)^ and affects 35 million people in the USA annually^(6)^. The most commonly used measures of food security in the USA focus on economic barriers to food access^(7)^. Respondents in numerous studies reported significant declines in household food security during the early stages of the pandemic as a result of widespread economic losses, restricted access and other challenges^(8,9)^. At the same time, existing food assistance programmes were modified in response to the challenges brought about by the pandemic, and new programmes were implemented to mitigate harms^(10)^. Additionally, there were policies enacted within the food system to minimise disruptions including the Defense Production Act to keep meat processing facilities open and operating^(11)^, as well as the declaration of grocery store, agriculture and food system workers as essential^(12)^. The US Department of Agriculture estimated no overall increase in food insecurity at the national level in 2020 and 2021^(5)^, potentially suggesting the effectiveness of these interventions, while pandemic and containment challenges continued^(10,13–18)^.

Economically focused food security measures and data were critical for informing policies during the pandemic^(19)^. Yet, the pandemic also demonstrated that food security is a complex, dynamic construct not limited to economic aspects^(20,21)^. For example, movement restrictions and lockdowns posed new challenges for physically getting to food sources, while supply chain disruptions, such as the closure of meat processing facilities due to COVID-19 outbreaks, reduced the availability of certain food items and led to shortages in stores. Such challenges were compounded by panic buying, particularly at the onset of the pandemic, when containment policies first went into effect^(22)^. Adapting Clay *et al*.’s (2023) Disaster Food Security Framework^(21)^, we highlight five dimensions of pandemic-related food security that could be affected by containment policies:

- Availability (e.g. is food present within an individual’s food environment, at the stores or other sources they prefer to use?)
- Accessibility/economic (e.g. is food affordable?)
- Accessibility/physical (e.g. can individuals physically reach food? Are there transportation, mobility, distance or safety limitations to accessing food sources?)
- Acceptability/preference (e.g. is food culturally acceptable, and does it meet at least basic taste preferences?)
- Agency
  - Infrastructure (e.g. does the person have the equipment needed to get and prepare the foods?)
  - Self-efficacy (e.g. does the person know how to prepare the foods?)

## Dimensions of containment policies during the pandemic

Multiple types of containment policies were enacted during the pandemic, including stay-at-home orders, closures of non-essential businesses and domestic and international travel restrictions. Based on the components of the Stringency Index in the Oxford COVID-19 Government Response Tracker (OxCGRT)^(23)^ (described below in methods), we focus on five types of policy: school closing, workplace closing, closing of public transport, stay-at-home requirements and restrictions on internal movement. The Stringency Index aggregates these policies into a single index; we consider them both jointly and separately.

Only a few studies so far have explored the ways different pandemic restrictions affected various health and economic outcomes. Most studies thus far using the Stringency Index have examined the association with COVID-19 cases and/or deaths^(24–29)^, hypothesising that countries with more stringent policies would have lower incident cases or death rates. Results have supported that hypothesis, demonstrating that countries and US states^(30,31)^ with more stringent policies were more successful at reducing the prevalence of COVID-19 than others, at least while the policies were in place.

## Conceptualising the relationship between food security and containment policies

Despite the beneficial impact of containment policies on the incidence of COVID-19 cases and deaths, they caused significant social disruption. There have been widespread concerns that these policies were detrimental to different aspects of mental and social well-being^(3,29,32–34)^, including potentially contributing to increases in food insecurity. No studies to date have specifically examined how containment policies may have impacted household food security status during the pandemic.

**Figure 1.**
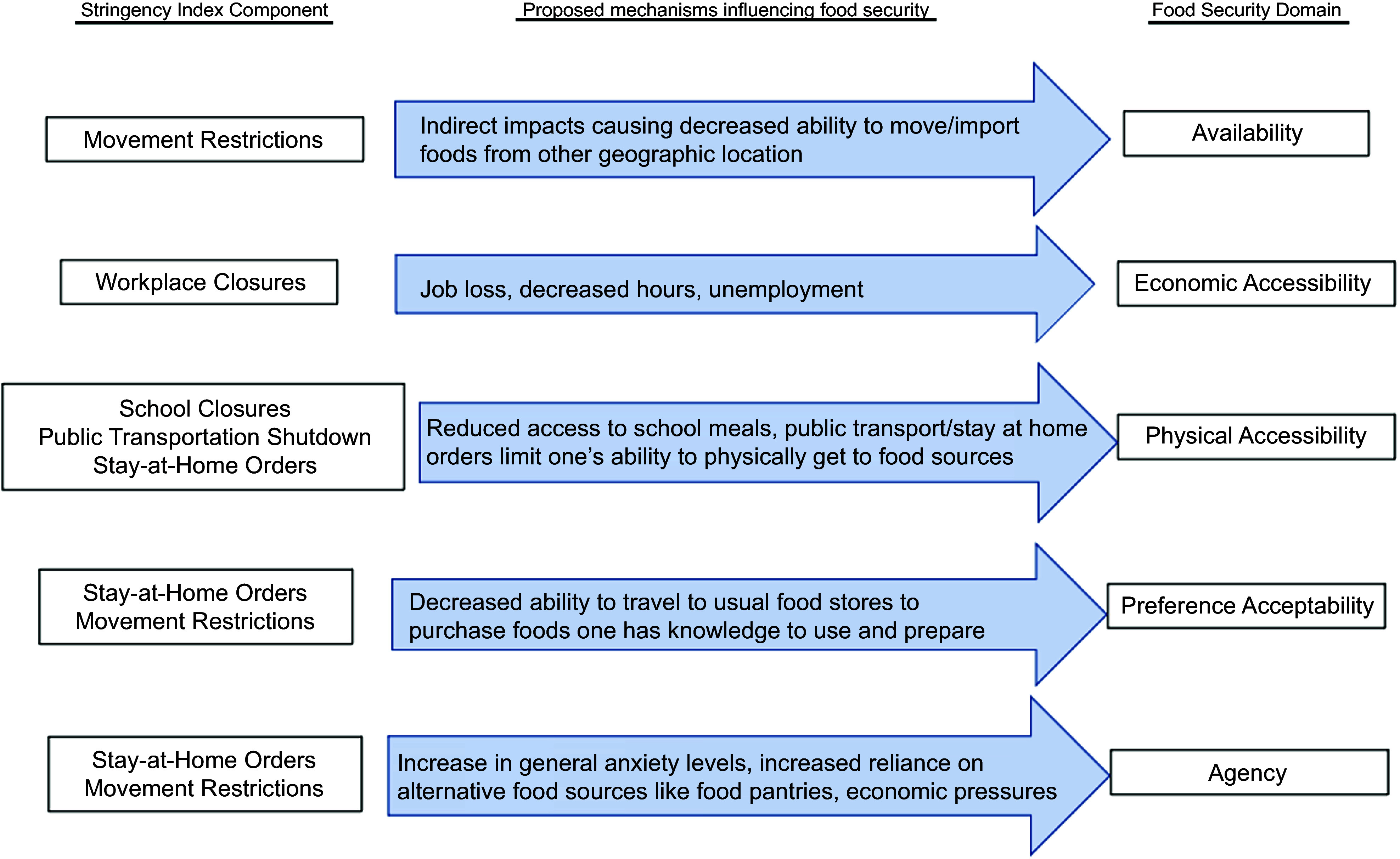
Framework linking food security domains to subcomponents of the Stringency Index score. The components of the Stringency Index are listed on the left, connected with arrows to the proposed domains of food security they may be linked with.

Figure 1 presents a conceptual framework suggesting potential ways in which the different types of containment policies may directly or indirectly affect the different food security domains. For example, as shown in the first row, movement restrictions (defined as restrictions on individual-level internal movement domestically between cities/regions) could indirectly impact food availability by restricting or delaying food imports or limiting the availability of certain food items in grocery stores and other food outlets.

Generally, the containment policies are expected to affect food security via indirect means and to have the largest impact on economic and physical accessibility domains. For example, when workplaces close, the projected impacts on economic access to food would be caused more directly by intermediate exposures to unemployment or increased food prices, which we suggest would in turn impact the economic accessibility food security domain. As noted above, mitigating factors can also include receipt of food assistance and other economic benefits.

This study builds on the above framework by examining (1) the relationship between household food security and state-level containment policies in the USA and (2) which domains of household food security may have been most affected by specific imposed COVID-19 containment policies. We hypothesised that US states with higher Stringency Index scores (i.e. stricter containment policies) would have higher rates of food insecurity across multiple domains, while controlling for other relevant factors that can also influence food security such as income, job loss and federal food assistance programme participation.

## Methods

### Household food security data

#### Overview and sampling

Food security and sociodemographic data were collected via the Coronavirus and Food Security Survey administered by the National Food Access and COVID research Team (NFACT) collaborative^(35)^. NFACT is a multi-state research collaborative that aimed to capture the extent of and broad determinants of household food security in the USA during the pandemic by implementing a survey at multiple time points, at the national, state and local levels^(36)^. This study combines data from surveys administered in July–August 2020 and April 2021. These time points shared comparable COVID-19 case rates and similar temporal distance from federal stimulus fund distribution but reflect different points in the pandemic policy trajectory. In both waves, participants were recruited either through convenience sampling (ten sites) or through online survey panels managed by Qualtrics in English and Spanish, using quotas to achieve a nationally representative sample based on gender, age and race (fourteen sites) (see Niles MT, 2021, for additional details on sampling and quota strategies)^(35)^. To be eligible for the survey, participants had to be 18 years or older, have lived in the USA since at least 1 January 2020 and read English or Spanish. Informed online consent was completed by all participants via Qualtrics prior to beginning the survey. The original NFACT study was conducted according to the guidelines of the Declaration of Helsinki and approved by the Institutional Review Board at each respective site^(35)^.

#### Measures

We used the US Household Food Security Survey Module: Six-Item Short Form^(7)^ (henceforth, HFSSM) to assess food insecurity. Respondents were asked about their household’s food security experiences in the 4 months prior to taking the survey as well as over the 12 months before the pandemic (March 2019–March 2020)^(7)^. Total food security score was calculated by summing the affirmative responses and categorising by high/marginal food security (0–1 points), low food security (2–4 points) and very low food security (5–6 points).

Building on the HFSSM, NFACT’s survey includes further questions (Table 1) that enable studying the food security domains of interest. While these categories are themselves multidimensional and some were addressed with multiple survey questions, for the purpose of this analysis, we chose one representative question for each domain, with four (availability, accessibility/economic, accessibility/physical, acceptability/preference) being validated to reflect these concepts^(21)^. Other measures collected in the NFACT survey included age, gender, race/ethnicity, whether there were children in the household, employment status, annual household income and education level of the individual respondents^(35)^. Questions with two response options (yes or no) were treated as binary variables (job loss, whether or not there are children in the household, enrolment in Supplemental Nutrition Assistance Program and Special Supplemental Nutrition Program for Women, Infants and Children, whether a household was food secure prior to the pandemic) and questions with multiple responses options were treated as categorical variables (food security status, age group, gender, race and income).

**Table 1.** Food security domains in the NFACT survey

Food security domain	Corresponding survey question
Availability	Participants were asked how often they experienced the following challenge related to getting food in the last 4 months: *‘Could not find AS MUCH food as I wanted to buy (food not in store)’* Responses were ranked on a Likert scale from 1 (never) to 4 (every time).
Accessibility: economic	In addition to the USDA HFSSM, participants were asked about their level of worry for their household related to getting food during the pandemic: *‘My household will lose so much income that we can’t afford enough food’* Responses were ranked on a Likert scale from 1 (not at all worried) to 6 (extremely worried).
Accessibility: physical	Participants were asked how often they experienced the following challenge related to getting food in the last 4 months: *‘Had to go to more places than usual to find the food my household wanted’* Responses were ranked on a Likert scale from 1 (never) to 4 (every time).
Acceptability: preference	Participants were asked how often they experienced the following challenge related to getting food in the last 4 months: *‘Could not find the types of food my household prefers to eat’* Responses were ranked on a Likert scale from 1 (strongly disagree) to 5 (strongly agree).
Agency: self-efficacy^*^	*‘The food pantry gives me foods I do not know how to prepare’* Responses were ranked on a Likert scale from 1 (strongly disagree) to 5 (strongly agree).
Agency: infrastructure^*^	*‘We do not have the kitchen equipment to safely store or re-heat meals’* [from school meals] Responses were ranked on a Likert scale from 1 (strongly disagree) to 5 (strongly agree).

NFACT, National Food Access and COVID research Team; USDA, US Department of Agriculture; HFSSM, Household Food Security Survey Module.

* These questions were only asked to a subset of participants to whom they applied.

### Containment policy data

To capture containment policies during the pandemic, the OxCGRT provides a tool for tracking such policies by geographic location (country and state levels)^(23)^. The tool tracks government policy responses across 180 countries, including the USA, both nationally and at the state level. The OxCGRT utilises data collected on twenty-three government response indicators categorised into four different policy areas: containment and closure policies, economic policies, health system policies and vaccine policies^(23)^. These twenty-three indicators are then aggregated to create four common indices, one being the Stringency Index. The Stringency Index has nine components: school closing, workplace closing, cancellation of public events, restrictions on gathering size, closing of public transport, stay-at-home requirements, restrictions on internal movement, restrictions on international travel and public information campaigns, which can be aggregated to create a Stringency Index score from 0 to 100 for an area at a point in time. The total possible score ranges from 0 to 100, with higher scores indicating more stringent policies.

State-level Stringency Index score data were downloaded from the OxCGRT website. The daily score for each state was averaged over the number of days during which NFACT data collection took place for the two time periods (time 1, 62 d; time 2, 30 d) to generate an overall average Stringency Index score for each state (see online Supplemental Table 1). Additionally, the Stringency Index was broken down into its nine subcomponents. The scores for each subcomponent are measured on an ordinal scale ranging from 0–2, 0–3 and 0–4 where 0 indicates no restrictions and higher numbers indicate the more stringent policies for each component. The scores are then reproportioned to range from 0 to 100 to create the total score^(37)^.

### Analysis

Descriptive statistics, such as means and frequencies, were used to examine participant sociodemographic characteristics. Multilevel, random effects (to account for the hierarchical structure of the data, with households as level 1 units sampled from different states (level 2 units)) ordinal logit models were utilised to analyse the association (1) between household food security status (using the US Department of Agriculture HFSSM categories: very low, low and high/marginal food security) and Stringency Index and (2) between each NFACT food security domain (availability, access/economic and access/physical, acceptability/preference, agency/self-efficacy + infrastructure) and the corresponding individual Stringency Index components as mapped in Figure 1 (school closures, workplace closures, public transportation shutdowns, stay-at-home orders and movement restrictions). The sample sizes for each food security domain vary due to different non-response rates for each question (e.g. if a respondent did not engage with the charitable food system, they were not required to answer questions about food-getting behaviours at food pantries). The study sample oversampled low-income households; thus, we applied survey weights constructed using census data (specifically from the American Community Survey data, see Niles MT, 2021, for further detail^(35)^) to reflect the national population in all analyses^(35)^. All models controlled for age, race/ethnicity, gender, income, whether there were children in the household, job status, food security status prior to the pandemic, participation in federal food assistance programmes (Supplemental Nutrition Assistance Program and/or Special Supplemental Nutrition Program for Women, Infants and Children) since the start of the pandemic and survey time point. All fifty states plus Washington, DC, were represented in survey responses. We ran two sensitivity analyses: (1) a sensitivity analysis where states with ten or fewer respondents were dropped; because the results were unchanged, we opted to report the results including all states; and (2) we ran a sensitivity analysis where the ‘job loss’ variable was not included given its potential duplicity with income, and the results were unchanged, and therefore ‘job loss’ remained in the model based on our conceptual framework. All analyses were conducted using Stata version 16^(38)^.

## Results

Table 2 provides participant sociodemographic details. At both time points, participant age was evenly distributed across categories 18–34, 35–54 and 55 and older, 50·1 % female, non-Hispanic White (61·6 %), with an annual income between $25 000 and $49 999 (33·2 %) and had children in their household (56·8 %). Most participants (65·7 %) were categorised as high/marginally food secure in the HFSSM.

**Table 2.** Weighted sociodemographic characteristics of National Food Access and COVID research Team (NFACT) survey participants in time 1 (July–August 2020) and time 2 (April 2021) combined (*n* 3071) and compared with the 2018 American Community Survey (ACS) data 5-year estimates

Characteristic	ACS	NFACT survey participants	
		*n* 3071	
		Mean	sd
Average state-level Stringency Index score, M (sd)	–	54·5	9·5
		*n*	%
Age, *n* (%)		(*n* 2997)	
18–34	23·1^*^	949	31·7
35–54	25·6	1011	33·7
55+	28·5	1037	34·6
Gender identity, *n* (%)			
Male	49·2	1341	43·7
Female	50·8	1711	55·7
Another gender		19	0·6
Race/ethnicity, *n* (%)			
Hispanic	17·8	524	17·1
Non-Hispanic White	72·7	1891	61·6
Non-Hispanic Black	12·7	420	13·7
Asian	5·4	129	4·2
Native American	1	4	0·1
Other/multiple	8·1	103	3·4
Annual household income in 2019, *n* (%)			
< $10 000	6·3	283	9·2
$10 000–$24 999	13·9	661	21·5
$25 000–$49 999	21·9	1018	33·2
$50 000–$74 999	17·5	336	10·9
$75 000–$99 999	12·5	239	7·8
$100 000+	27·9	534	17·4
Children in the household, *n* (%)			
Yes	–	1732	56·8
No	–	1207	39·6
Education, *n* (%)			
Some high school	–	697	23·0
High school/GED	26·9^**^	662	21·8
Some college	20	376	12·4
Associates/tech school/apprenticeship	8·6	758	25·0
Bachelor’s degree	20·3	540	17·8
Lost job (since the start of the pandemic)			
Yes	–	476	23·0
No	–	1592	77·0
USDA food security status			
High/marginal food security	–	2018	65·7
Low food security	–	605	19·7
Very low food security	–	448	14·6

USDA, US Department of Agriculture.

* This ACS category did not line up with the NFACT survey age groups. Therefore, this category was created by calculating two-fifths of the ACS age category 15–19 and adding it to categories 20–24, 25–29 and 30–34, providing an estimate of 18–34. Thus, the ACS age percentages do not add up to 100 %.

** Combined as ‘high school or less’.

At time 1, the aggregated average state-level Stringency Index score was 50·3 (sd 11·8) (range: 21·3–79·7) points, while at time 2, it was 38·4 (sd 12·6) (range: 9·3–66·9) points. This difference was expected given that some COVID-19 policies had been relaxed by this time (April 2021).

Table 3 reports the results from the multilevel ordinal logit regression models. The exposure variables appear on the left side, and the outcome variable for each model is household food security status. First, the overall associations between the average state-level Stringency Index score and household food security status are presented. Overall, the average state-level Stringency Index score was not associated with household food security status (OR 1·00; CI 0·99, 1·01; *P* = 0·530) after controlling for all covariates listed above. Lastly, we present separate multilevel models for each of the eleven theorised relationships between types of containment policy and aspects of food security. No significant associations were found. We also explored these relationships in a similar fashion at both time points individually, and the results were consistent with the results from the full sample.

**Table 3.** Multilevel models^*^ for individual Stringency Index components and individual food security domains from the National Food Access and COVID research Team survey across time 1 (July–August 2020) and time 2 (April 2021), based on the conceptual framework in Figure 1. *n* 3071^†^

Model 1: Overall associations	OR	95 % CI
1a. Food insecure since the pandemic (Yes/No)		
Time 1		
Average Stringency Index score	1·00	0·98, 1·02
Time 2		
Average Stringency Index score	1·01	0·99, 1·03
1b. USDA 3 category food security status	OR	95 % CI
Time 1		
Average Stringency Index score	1·01	0·99, 1·03
Time 2		
Average Stringency Index score	0·99	0·97, 1·00
Model 2: Individual domains (time 1 and time 2 combined)	OR	95 % CI
2a. Availability Could not find AS MUCH food as I wanted to buy (food not in store) (*n* 1909)		
Movement restrictions	1·08	0·93, 1·27
2b. Accessibility: economic My household will lose so much income that we can’t afford enough food (*n* 1876)		
Workplace closures	1·07	0·92, 1·25
2c. Accessibility: physical Had to go to more places than usual to find the food my household wanted (*n* 1874)		
School closures	1·31	0·92, 1·86
Public transport shutdown	0·87	0·73, 1·04
Stay-at-home orders	1·05	0·82, 1·35
2d. Acceptability: preference Could not find the types of food my household prefers to eat (*n* 1899)		
Stay-at-home orders	1·01	0·80, 1·27
Movement restrictions	1·01	0·87, 1·18
2e. Agency: self-efficacy^†^ The food pantry gives me foods I do not know how to prepare (*n* 395)		
Stay-at-home orders	1·05	0·63, 1·76
Movement restrictions	1·04	0·75, 1·45
2f. Agency: infrastructure^†^ We do not have the kitchen equipment to safely store or re-heat meals (*n* 226)		
Stay-at-home orders	1·59	0·75, 3·35
Movement restrictions	1·12	0·68, 1·84

USDA, US Department of Agriculture.

* Models were controlled for age, race, income, whether the household included children, federal food assistance programme participation, job status and time point.

† The *N*s varied across model due to different non-response rates for each question and that some questions were only asked to a subset of participants to whom it applied.

## Discussion

This research, using a newly developed framework linking stringency policies and food security, suggests that while food insecurity across all domains was a significant problem during the studied phases of the pandemic, it was not associated with these containment measures. Alternatively, the potential impacts may have been successfully mitigated by policies that expanded food assistance programmes and supported food system operations^(10–12)^. We found no relationship between the Stringency Index score and food security measures, both as a whole and when examining the individual components of each. All models controlled for relevant individual and household-level characteristics, such as age, race, income, whether there were children in the household, job status, food security status prior to the pandemic and survey time point. While these findings should be considered preliminary, pending further confirmatory research with larger sample sizes, especially across small population states, they provide encouraging results, as these policies were originally implemented to safeguard public health, a goal they have effectively achieved^(10,13–18)^.

One possible explanation for the current null findings is that supportive policies and interventions may have buffered the impact of closure and containment measures on food security. These include expanded unemployment benefits, stimulus checks, expanded child tax credit in 2021, expansion of federal programmes such as the Supplemental Nutrition Assistance Program and the Special Supplemental Nutrition Program for Women, Infants and Children benefits, introduction of new programmes such as the Pandemic Electronic Benefits Transfer (P-EBT) and direct food assistance and mutual aid^(19,39,40)^. Evidence suggests that such interventions may have mitigated food insecurity during the pandemic^(10,13–16,18)^. Furthermore, other policies were explicitly put in place with a goal to minimise disruptions to the food system. These include an executive order invoking the Defense Production Act to keep meat processing facilities open and operating^(11)^, as well as the declaration of grocery store, agriculture and food system workers as essential^(12)^. We did not include these potentially mitigating factors in this analysis given their collinearity with food security and the complexity in attempting to infer causality based on any associations between food policies and food security status. It is possible that states with higher Stringency Index scores were more proactive in their policies and programmes than other states. For example, states with stronger social welfare orientation may have been both more likely to enact containment policies and to support interventions to address poverty, food insecurity or mitigate the potential impacts of containment policies. Thus, one potential explanation for our findings is that even though the containment policies would otherwise have harmed food security, these interventions were geographically associated with containment policies and sufficient in magnitude to mitigate their unintended, negative impacts. Further research is needed to assess this possibility.

The social and policy environment in which this study was conducted is complex, and it was not possible to include variables capturing all of the variability in baseline conditions, pandemic outcomes and economic factors across time. In particular, state differences in baseline food insecurity and baseline food security interventions could have affected the results. Such policies could vary by state political affiliation, although associations between political affiliation and COVID-19 cases, deaths and testing fluctuated throughout the pandemic in complex ways^(41)^.

### Strengths and limitations

The present study draws strength from its basis in combined measures collected at two time points, using a sample selected for generalisability to the US population. Because the sample was not limited to those expected to have particular vulnerability to food insecurity, it enables estimates for the general US population. In addition to being the first study to examine the relationship between household food security and containment policies across US states, this study also introduces a novel framework illustrating how five domains of food security may be linked with containment policies. This framework can be used elsewhere both to further understand the different facets of food security of concern during a pandemic and to explore the effects of containment policies.

The results should be interpreted with several limitations in mind. First, we did not have the data needed to evaluate the intermediate pathways implied by this framework; this could be studied in future research. Second, sampling bias may limit generalisability to the US population, particularly those without internet access and non-English speakers. Third, there is a risk of recall bias. The study also does not account for further specific state-level characteristics that may confound the relationship between food security and Stringency Index. Although the analysis in this study controlled for current job status, we did not include data on the use of other pandemic relief benefits such as unemployment benefits because this was only collected at time 2; we also did not include other interventions that could mitigate outcomes such as usage of direct food assistance like food pantries. The decision to select only one NFACT survey question for each aspect of the food security framework means that the survey questions do not comprehensively reflect all aspects of the concepts to which they are applied. Additionally, the NFACT survey questions related to the agency require further validation to ensure they capture the domains of food security to which they are applied. It is also possible that the Stringency Index did not fully capture the aspects of containment policies that could have influenced food security and state-level compliance with restrictions; further, policy compliance was not assessed^(42)^. Lastly, there were twelve states at time 1 and eleven states at time 2 from which ten or fewer respondents were sampled, limiting the state-level variation and representativeness in the outcome. Nonetheless, the results remained robust despite low sample sizes in some states, as sensitivity analysis excluding respondents from these states produced identical results to those from the full sample.

### Conclusions

In this study, there was no identified relationship between state-level COVID-19 containment policies and household food security measures overall or in any of the studied food security domains (i.e. availability, economic accessibility, physical accessibility, accessibility in meeting preferences and agency in terms of both infrastructure and self-efficacy to preparing available foods). These findings were unexpected. Further research is needed using alternative measures and time points, including impacts at different income levels and further data about mitigation interventions, to add to the understanding of these potential mechanisms. The observed lack of association between containment policies and food security is potentially the result of other mitigating policies and strategies implemented across the food system to minimise such disruptions. The containment policies implemented during COVID-19 were introduced with the intent to limit infection spread and save lives; nonetheless, they created trade-offs in social and economic well-being. In the future, policymakers may again be faced with challenging decisions about implementing similar policies to limit exposure risks. While containment policies should not be adopted lightly, this multilevel analysis provides evidence, albeit preliminary, supporting their adoption, along with other food system and security mitigation interventions, when warranted by public health risks.

## Supporting information

Sundermeir et al. supplementary material Sundermeir et al. supplementary material

## Supplementary material

For supplementary material/s referred to in this article, please visit https://doi.org/10.1017/S1368980024002696.

## References

[1] Our World in Data (2021) COVID-19: Stringency Index. https://ourworldindata.org/grapher/covid-stringency-index (accessed 09 September 2021).

[2] Food Security and COVID-19 (2021) World Bank. https://www.worldbank.org/en/topic/agriculture/brief/food-security-and-covid-19 (accessed 09 September 9 2021).

[3] Niles MT , Bertmann F , Belarmino EH et al. (2020) The early food insecurity impacts of COVID-19. Nutrients 12, 2096. 32679788 10.3390/nu12072096 PMC7400862

[4] Aday S & Aday MS (2020) Impact of COVID-19 on the food supply chain. Food Qual Saf 4, 167–180. doi: 10.1093/fqsafe/fyaa024. PMC7499675 40504224

[5] United States Department of Agriculture Economic Research Service Website Food Security in the U.S.: Key Graphs and Statistics. Updated September 2021. https://www.ers.usda.gov/topics/food-nutrition-assistance/food-security-in-the-u-s/key-statistics-graphics/#insecure (accessed March 2022).

[6] The United States Department of Agriculture Website Food Security in the US: Key Statistics & Graphics. Updated September 8, 2021. https://www.ers.usda.gov/topics/food-nutrition-assistance/food-security-in-the-us/key-statistics-graphics.aspx (accessed 16 September 2021).

[7] Economic Research Service, USDA (2012) U.S. Household Food Security Survey Module: Six-Item Short Form. September 2012. https://www.ers.usda.gov/topics/food-nutrition-assistance/food-security-in-the-us/survey-tools#six (accessed 14 April 2021).

[8] Nicola M , Alsafi Z , Sohrabi C et al. (2020) The socio-economic implications of the coronavirus pandemic (COVID-19): a review. Int J Surg 78, 185–193. 32305533 10.1016/j.ijsu.2020.04.018 PMC7162753

[9] Siddiqi SM , Cantor J , Dastidar MG et al. (2021) SNAP participants and high levels of food insecurity in the early stages of the COVID-19 pandemic. Public Health Rep 136, 457–465. 33789530 10.1177/00333549211007152 PMC8203047

[10] Caspi C , Seligman H & Berge J (2022) COVID-19 Pandemic-Era Nutrition Assistance: Impact and Sustainability. Health Affairs Health Policy Brief, May 5, 2022. doi: 10.1377/hpb20220330.534478.

[11] The Hill (2020) Trump uses Defense Production Act to Order Meat Processing Plants to Stay Open. https://thehill.com/homenews/administration/495175-trump-uses-defense-production-act-to-order-meat-processing-plants-to/ (accessed March 2024).

[12] CDC (2020) Interim List of Categories of Essential Workers Mapped to Standardized Industry Codes and Titles. Center for Disease Control and Prevention Website. https://archive.cdc.gov/#/details?url=https://www.cdc.gov/vaccines/covid-19/categories-essential-workers.html (accessed March 2024).

[13] Bovell-Ammon A , McCann NC , Mulugeta M et al. (2022) Association of the expiration of child tax credit advance payments with food insufficiency in US households. JAMA Netw Open 5, e2234438. 36269356 10.1001/jamanetworkopen.2022.34438 PMC9587477

[14] Shafer PR , Gutiérrez KM , Ettinger de Cuba S et al. (2022) Association of the implementation of child tax credit advance payments with food insufficiency in US households. JAMA Netw Open 5, e2143296. 35024837 10.1001/jamanetworkopen.2021.43296 PMC8759005

[15] Adams E , Brickhouse T , Dugger R et al. (2022) Patterns of food security and dietary intake during the first half of the child tax credit expansion. Health Aff (Millwood) 41, 680–688. 35500174 10.1377/hlthaff.2021.01864 PMC9614666

[16] Waxman E , Salas J , Gupta P et al. (2022) Food Insecurity Trended Upward in Midst of High Inflation, Fewer Supports. Published September 2022. https://www.urban.org/sites/default/files/2022-09/HRMS%20Food%20Insecurity%20Brief_0.pdf (accessed November 2022).

[17] Raifman J , Bor J & Venkataramani A (2021) Association between receipt of unemployment insurance and food insecurity among people who lost employment during the COVID-19 pandemic in the United States. JAMA Netw Open 4, e2035884. 33512519 10.1001/jamanetworkopen.2020.35884 PMC7846943

[18] Bryant A & Follett L (2022) Hunger relief: a natural experiment from additional SNAP benefits during the COVID-19 pandemic. Lancet Reg Health Am 10, 100224. 35284905 10.1016/j.lana.2022.100224 PMC8901427

[19] USDA Food and Nutrition Service FNS Responds to COVID-19. The U.S. Department of Agriculture Food and Nutrition Service Website. https://www.fns.usda.gov/coronavirus. (accessed December 2021).

[20] Food Security Policy Brief (2006) Food and Agriculture Organization’s Website. Issue 2, Published June 2006. https://www.fao.org/fileadmin/templates/faoitaly/documents/pdf/pdf_Food_Security_Cocept_Note.pdf (accessed April 2023).

[21] Clay LA , Koyratty N , Rogus S et al. (2023) A mixed-methods approach to the development of a disaster food security framework. J Acad Nutr Diet 123, S46–S58. 37730306 10.1016/j.jand.2023.05.005

[22] Taylor S (2021) Understanding and managing pandemic-related panic buying. J Anxiety Disord 78, 102364. 33517219 10.1016/j.janxdis.2021.102364

[23] BSG (2020) COVID-19 Government Response Tracker. https://www.bsg.ox.ac.uk/research/research-projects/covid-19-government-response-tracker (accessed 27 June 2021).

[24] Wang X , Chen C , Du Y et al. (2021) Analysis of policies based on the multi-fuzzy regression discontinuity, in terms of the number of deaths in the coronavirus epidemic. Healthcare (Basel) 9, 116. 33499030 10.3390/healthcare9020116 PMC7912350

[25] Cao Y , Hiyoshi A & Montgomery S (2020) COVID-19 case-fatality rate and demographic and socioeconomic influencers: worldwide spatial regression analysis based on country-level data. BMJ Open 10, e043560. 10.1136/bmjopen-2020-043560 PMC7640588 33148769

[26] Chen SX , Lam BCP , Liu JH et al. (2021) Effects of containment and closure policies on controlling the COVID-19 pandemic in East Asia. Asian J Soc Psychol 24, 42–47. 33821141 10.1111/ajsp.12459 PMC8014465

[27] Achuo E (2020) How efficient are government stringency responses in curbing the spread of the COVID-19 pandemic? Int J Res Innovation Soc Sci 4, 629–635.

[28] Sulyok M & Walker MD (2021) Mobility and COVID-19 mortality across Scandinavia: a modeling study. Travel Med Infect Dis 41, 102039. 33785456 10.1016/j.tmaid.2021.102039 PMC7999697

[29] Lee JH , Lee H , Kim JE et al. (2021) Analysis of personal and national factors that influence depression in individuals during the COVID-19 pandemic: a web-based cross-sectional survey. Global Health 17, 3. 33402174 10.1186/s12992-020-00650-8 PMC7783293

[30] The New York Times (2020) States That Imposed Few Restrictions Now Have the Worst Outbreaks. Published November 2020. https://www.nytimes.com/interactive/2020/11/18/us/covid-state-restrictions.html (accessed March 2024).

[31] BSG (2020) Variation in US States’ Responses to COVID-19. BSG Working Paper Series. Published December 2020. https://www.bsg.ox.ac.uk/sites/default/files/2020-12/BSG-WP-2020-034-v2_0.pdf (accessed August 2022).

[32] Bertoldo J , Wolfson JA , Sundermeir SM et al. (2022) Food insecurity and delayed or forgone medical care during the COVID-19 pandemic. Am J Public Health 112, 776–785. 35417213 10.2105/AJPH.2022.306724 PMC9010899

[33] Wolfson JA & Leung CW (2020) Food insecurity during COVID-19: an acute crisis with long-term health implications. Am J Public Health 110, 1763–1765. 32970451 10.2105/AJPH.2020.305953 PMC7662000

[34] Galea S , Merchant RM & Lurie N (2020) The mental health consequences of COVID-19 and physical distancing: the need for prevention and early intervention. JAMA Intern Med 180, 817–818. 32275292 10.1001/jamainternmed.2020.1562

[35] Niles MT , Beavers AW , Clay LA et al. (2021) A multi-site analysis of the prevalence of food insecurity in the United States, before and during the COVID-19 pandemic. Curr Dev Nutr 5, nzab135. 34934898 10.1093/cdn/nzab135 PMC8677520

[36] Niles MT , Neff R , Biehl E et al. (2020) Food Access, Food Security during COVID-19 Survey-Version 2.1. Harvard Dataverse. 3. https://dataverse.harvard.edu/dataset.xhtml?persistentId=doi:10.7910/DVN/RQ6NMG (accessed August 2022).

[37] Hale T , Anania J , Mello BA et al. (2022) ‘Variation in Government Responses to COVID-19’ Version 13.0. Blavatnik School of Government Working Paper. 11 March 2022. https://github.com/OxCGRT/covid-policy-tracker/blob/master/documentation/index_methodology.md (accessed August 2022).

[38] StataCorp (2019) *Stata Statistical Software: Release 16*. College Station, TX: StataCorp LLC.

[39] United States Congress (2021) American Rescue Plan Act of 2021. United States Congress Website. https://www.congress.gov/bill/117th-congress/house-bill/1319/text (accessed 14 April 2021).

[40] United States Congress (2019) Families First Coronavirus Response Act (FFCRA) American Rescue Plan Act of 2020. United States Congress Website. https://www.congress.gov/bill/116th-congress/house-bill/1319/text (accessed 05 April 2021).

[41] Neelon B , Mutiso F , Mueller NT et al. (2021) Associations between governor political affiliation and COVID-19 cases, deaths, and testing in the U.S. Am J Prev Med 61, 115–119. 33775513 10.1016/j.amepre.2021.01.034 PMC8217134

[42] Mukerjee S , Chow CM & Li M (2021) Mitigation strategies and compliance in the COVID-19 fight; how much compliance is enough? PLoS One 16, e0239352. 34370739 10.1371/journal.pone.0239352 PMC8351990

