# Supplementary material for: US state-level containment policies not associated with food insecurity changes during the early COVID-19 pandemic: a multilevel analysis
Source: Public Health Nutr. 2025 Jan 23;28(1):e37. doi: 10.1017/S1368980024002696 (PMC11822581; doi:10.1017/S1368980024002696)
Supplement: Sundermeir et al. supplementary material [file S1368980024002696sup001.docx]

**Supplemental Table 1**: Number of NFACT Survey Respondents and Stringency Index Scores at the National and State Level at Time 1 (July-August 2020) and Time 2 (April 2021)

| **Location** | **N**  **Time 1** | **Stringency Index Score at Time 1** | **N**  **Time 2** | **Stringency Index Score at Time 2** |
| --- | --- | --- | --- | --- |
| The United States Overall | 1,415 | 67.7 | 1,656 | 56.9 |
| Alaska | 2 | 46.6 | 4 | 43.5 |
| Alabama | 18 | 35.2 | 31 | 18.5 |
| Arkansas | 12 | 45.4 | 12 | 26.9 |
| Arizona | 13 | 45.4 | 33 | 21.7 |
| California | 171 | 61.2 | 180 | 56.6 |
| Colorado | 28 | 50.5 | 28 | 35.2 |
| Connecticut | 22 | 56.8 | 27 | 48.9 |
| Washington D.C. | 4 | 61.3 | 7 | 53.7 |
| Delaware | 5 | 62.8 | 0 | 44.0 |
| Florida | 148 | 58.4 | 138 | 35.9 |
| Georgia | 54 | 47.6 | 54 | 27.3 |
| Hawaii | 7 | 76.4 | 7 | 66.9 |
| Iowa | 10 | 26.4 | 7 | 19.3 |
| Idaho | 6 | 44.0 | 4 | 32.4 |
| Illinois | 60 | 49.0 | 56 | 43.0 |
| Indiana | 24 | 43.0 | 21 | 33.3 |
| Kansas | 6 | 42.7 | 9 | 26.7 |
| Kentucky | 18 | 53.4 | 21 | 30.2 |
| Louisiana | 17 | 53.5 | 20 | 40.4 |
| Massachusetts | 18 | 59.7 | 28 | 57.2 |
| Maryland | 20 | 53.4 | 22 | 43.5 |
| Maine | 6 | 74.9 | 3 | 43.5 |
| Michigan | 27 | 54.6 | 40 | 46.3 |
| Minnesota | 23 | 53.2 | 16 | 51.9 |
| Missouri | 21 | 36.3 | 37 | 37.5 |
| Mississippi | 9 | 45.0 | 18 | 22.2 |
| Montana | 3 | 47.9 | 3 | 28.6 |
| North Carolina | 51 | 56.6 | 65 | 43.5 |
| North Dakota | 0 | 34.7 | 5 | 18.3 |
| Nebraska | 11 | 40.3 | 11 | 22.3 |
| New Hampshire | 2 | 40.7 | 8 | 38.9 |
| New Jersey | 33 | 53.8 | 48 | 47.6 |
| New Mexico | 5 | 79.7 | 11 | 45.6 |
| Nevada | 16 | 50.7 | 21 | 39.2 |
| New York | 166 | 70.8 | 142 | 48.5 |
| Ohio | 52 | 60.0 | 77 | 42.6 |
| Oklahoma | 8 | 30.8 | 15 | 38.0 |
| Oregon | 15 | 53.1 | 17 | 55.7 |
| Pennsylvania | 66 | 47.1 | 86 | 27.6 |
| Rhode Island | 5 | 63.3 | 6 | 56.5 |
| South Carolina | 17 | 38.8 | 16 | 25.0 |
| South Dakota | 4 | 21.3 | 8 | 9.3 |
| Tennessee | 31 | 47.3 | 28 | 31.5 |
| Texas | 102 | 52.9 | 112 | 39.4 |
| Utah | 2 | 41.5 | 10 | 19.9 |
| Virginia | 27 | 48.8 | 34 | 38.0 |
| Vermont | 0 | 58.8 | 2 | 56.5 |
| Washington | 23 | 53.7 | 29 | 57.3 |
| Wisconsin | 49 | 37.5 | 25 | 42.1 |
| West Virginia | 2 | 55.3 | 13 | 42.8 |
| Wyoming | 0 | 43.0 | 2 | 38.0 |
